# Determination of the ED_50_ and ED_95_ of intrathecal ropivacaine for labor analgesia in multiparous women: A prospective, sequential allocation study

**DOI:** 10.1097/MD.0000000000051174

**Published:** 2026-10-23

**Authors:** ZhiRong Yan, PeiHan Xiao, LiYan Miao, XiaoFen Chen, LongXin Zhang, SuJing Zhang

**Affiliations:** a Department of Anesthesiology, Fujian Maternity and Child Health Hospital, College of Clinical Medicine for Obstetrics & Gynecology and Pediatrics, Fujian Medical University, Fuzhou, Fujian Province, China.

**Keywords:** Dose-Response Relationship, Labor Pain, Ropivacaine, Spinal Anesthesia

## Abstract

With the implementation of China’s two-child policy,the number of multiparous women has increased significantly. However, most studies on labor analgesia have focused on nulliparous women, and research on analgesia for multiparous women remains relatively limited. This study aimed to determine the median effective dose (ED_50_) and 95% effective dose (ED_95_) of intrathecal ropivacaine for labor analgesia in multiparous women. Thirty multiparous parturients were enrolled. The initial dose of intrathecal ropivacaine was set at 3.0 mg. For each subsequent participant, the dose was adjusted by 0.3 mg increments or decrements based on the analgesic response of the previous subject. Effective analgesia was defined as a numerical rating scale (NRS) score ≤ 3 achieved within 10 minutes after intrathecal administration. If analgesia was effective, the ropivacaine dose for the subsequent patient in that group was decreased by 0.3 mg. Conversely, if analgesia was ineffective, the dose for the next patient in that group was increased by 0.3mg. For women with ineffective analgesia 10 minutes after intrathecal study drug administration,10 mL of 0.08% ropivacaine combined with 0.4 μg/mL sufentanil was administered epidurally as rescue analgesia. The ED_50_ and ED_95_ of intrathecal ropivacaine determined by Probit regression were 2.16 mg (95% CI: 1.83–2.39 mg) and 2.76 mg (95% CI: 2.49–3.02 mg), respectively. The incidence of adverse events during effective spinal analgesia was low. No cases of bradycardia, respiratory depression, hypotension, or motor block were observed. The ED_50_ and ED_95_ of intrathecal ropivacaine for labor analgesia in multiparous women during spontaneous latent phase labor were 2.16 mg and 2.76 mg, respectively. (www.chictr.org.cn, registration number: ChiCTR2100054379).

## 1. Introduction

The implementation of the two‑child family policy in China has led to a significant increase in the number of multiparous women. Labor in multiparous women is often characterized by a more rapid onset and escalation of pain after the initiation of uterine contractions, greater contraction intensity, and a shorter overall duration of labor,^[1–3]^ These features call for analgesia that acts more quickly and with greater efficacy. As a result, there is a pressing need for neuraxial analgesia that provides both rapid onset and enhanced effectiveness to ensure maternal comfort and safety

Among the current options for labor analgesia, neuraxial techniques such as epidural and combined spinal-epidural (CSE) methods are the most effective for pain management.^[4–6]^ CSE analgesia has gained increasing preference due to its faster onset, superior pain relief, reduced requirement for epidural top‑ups, lower drug exposure to both mother and fetus, and higher patient satisfaction.^[4,7,8]^ The use of ropivacaine in CSE has increased, partly owing to its favorable motor block profile.^[4,9]^ While the addition of intrathecal sufentanil to ropivacaine can enhance analgesic efficacy, it is also associated with potential risks of fetal distress and pruritus.^[5,10]^ To minimize the risk of opioid-related adverse effects, our institution adopted single intrathecal ropivacaine without the addition of sufentanil as an adjuvant. The ED_50_ of intrathecal ropivacaine for labor analgesia has been reported as 1.95 mg in nulliparous women.^[11]^ Previous study has also shown that multiparous women exhibit a higher epidural ropivacaine EC_50_ compared with nulliparous women.^[12]^ Whether the ED_50_ of intrathecal ropivacaine is similarly elevated in multiparous women, however, remains unclear. To date, the ED_50_ and ED_95_ of intrathecal ropivacaine for labor analgesia in multiparous women remain undetermined.

This prospective study aims to determine the ED_50_ and ED_95_ of intrathecal ropivacaine for labor analgesia in multiparous women using the up-down sequential allocation method, thereby providing an evidence-based foundation for optimizing analgesic dosing during the latent phase of labor in this growing patient group.

## 2. Materials and methods

### 2.1. Study design and ethical approval

This was a prospective, sequential allocation, up-and-down study designed to determine the ED_50_ and ED_95_ of intrathecal ropivacaine for labor analgesia in multiparous women. The study was conducted at Fujian Maternity and Child Health Hospital, a tertiary specialized hospital with an annual delivery volume of over 20,000. Ethical approval was granted by the Institutional Ethics Committee (Approval No. 2021KLR09041), and the study was prospectively registered with the Chinese Clinical Trial Registry on December 16, 2021 (Registration Identifier: ChiCTR2100054379) prior to participant enrollment. All participants provided written informed consent before any study procedures were initiated.

### 2.2. Participants

Multiparous women meeting the following criteria were eligible for inclusion: American Society of Anesthesiologists (ASA) physical status II, a healthy singleton pregnancy of at least 37 weeks of gestation, cervical dilation between 1 and 3 cm, and painful contractions requiring analgesia. The study primarily evaluated the effect of the first intrathecal dose administered during the latent to early active phase (cervical dilation ≤ 3 cm), a period characterized by relatively stable pain conditions suitable for determining the ED_50_ and ED_95_. Exclusion criteria comprised: refusal to participate, contraindications to neuraxial (spinal/epidural) puncture, known allergy to local anesthetics, morbid obesity (body mass index ≥ 35 kg/m^2^), preexisting neurological disease, prior uterine surgery, prostaglandin or oxytocin use for induction/augmentation, multiple pregnancy, or severe obstetric complications such as preeclampsia or fetal distress.

### 2.3. Procedures

Upon arrival in the delivery room, all participants underwent standard physiological monitoring, including non‑invasive blood pressure, pulse oximetry, and continuous fetal heart rate monitoring. Following informed consent, a peripheral intravenous catheter was placed. In accordance with our institutional protocol, which does not require a preload, no fluid bolus was administered before performing the neuraxial block. With the patient in the left lateral decubitus position, the epidural space at the L3‑4 interspace was located using an 80‑mm, 16‑gauge Tuohy needle via the loss‑of‑resistance technique with saline. A 12‑cm, 25‑gauge Whitacre spinal needle was then advanced through the epidural needle. Upon confirmation of free cerebrospinal fluid flow, the assigned dose of ropivacaine, diluted with cerebrospinal fluid to a volume of 3 mL (a volume sufficient to ensure adequate block height and avoid insufficient analgesia due to low block level), was injected intrathecally. The spinal needle was then withdrawn, and an epidural catheter was threaded 4 to 5 cm into the epidural space and secured. The patient was subsequently placed in the supine position.

The Dixon sequential method was employed in this study. A 3 mg/mL ropivacaine working solution was prepared in advance by diluting 3 mL of 10 mg/mL ropivacaine stock solution with 0.9% sodium chloride injection to a final volume of 10 mL. For each dose adjustment, 0.1 mL of the working solution was accurately aspirated, ensuring a consistent adjustment step of 0.3 mg per change. Based on clinical experience, an initial dose of 3.0 mg was administered to the first participant. An anesthesia nurse blinded to the ropivacaine dose evaluated the analgesic effect using a numerical rating scale (NRS), where 0 indicated no pain, 1 to 3 mild pain, 4 to 6 moderate pain, and 7 to 10 severe pain. Effective analgesia was defined as an NRS score ≤ 3. If analgesia was effective, the ropivacaine dose for the next participant was reduced by 0.3 mg; conversely, if analgesia was ineffective, the dose was increased by 0.3 mg. For participants who did not achieve effective analgesia 10 minutes after intrathecal administration, 10 mL of 0.08% ropivacaine combined with 0.4 μg/mL sufentanil was administered epidurally as rescue analgesia. Fetal heart rate was continuously monitored following the procedure. Maternal blood pressure (BP) and heart rate (HR) were recorded every 5 minutes for 30 minutes. Motor block was assessed concurrently using a modified Bromage scale (0 = no block; 1 = unable to raise extended leg, able to move knee and foot; 2 = unable to raise extended leg or move knee, able to move foot; 3 = complete motor block of the limb).

### 2.4. Study outcomes

The primary outcomes were the ED_50_ and ED_95_ of intrathecal ropivacaine for labor analgesia in multiparous women. Secondary outcomes included maternal and neonatal outcomes from the time of intrathecal injection until the first request for supplemental epidural analgesia. Maternal outcomes comprised bradycardia (heart rate < 50 bpm, treated with intravenous atropine 0.5 mg), hypotension (systolic blood pressure decrease > 20% from baseline or < 90 mm Hg, managed with intravenous fluid, left lateral tilt, and intravenous ephedrine as needed), motor block (modified Bromage score > 0), urinary retention, nausea/vomiting, and cesarean delivery. Neonatal outcomes included fetal bradycardia (fetal heart rate < 110 bpm) and Apgar scores at 1 and 5 minutes. Demographic and baseline clinical data collected included maternal age, weight, height, gestational age, pre‑analgesia NRS score, and cervical dilation at analgesia initiation. Participants with effective analgesia were classified as the effective group, while those requiring rescue medication formed the ineffective group. Between-group comparisons were performed for demographic characteristics, maternal outcomes, and neonatal outcomes.

### 2.5. Statistical analysis

Simulation studies have indicated that 30 participants per group are sufficient to observe at least 6 pairs of reversals in sequence using the up‑and‑down allocation method.^[13,14]^ Accordingly, 30 patients were included in this study. Data were analyzed with SPSS 26.0 (IBM Corp.) and are expressed as mean ± standard deviation (SD), median (interquartile range), or number (percentage), as appropriate. The ED_50_, ED_95_ and their respective 95% confidence intervals (CIs) for the sequential data were calculated using Probit regression analysis. Sequence response diagrams and dose-response curves were constructed to illustrate the up-and-down sequences and to estimate the effective doses. Continuous variables were compared with independent‑samples t‑tests or the Mann‑Whitney U test, as appropriate. Categorical variables were analyzed with the Chi‑square test or Fisher’s exact test. A P‑value < 0.05 was considered statistically significant.

## 3. Results

### 3.1. Patient demographics and obstetric characteristics

A total of 36 patients were recruited between January 2022 and March 2022. Of these, 4 did not meet the inclusion criteria and 2 declined to participate. Data from 30 participants were included in the final statistical analysis (Fig. 1). Demographic and obstetric characteristics are summarized in Table 1. The groups were comparable in age, weight, height, gestational age, body mass index, and baseline cervical dilation.

**Table 1:** Patient demographics and obstetric characteristics.

Variables	Effective group (n = 17)	Ineffective group (n = 13)	*P* value
Age,yr	31.2 ± 5.0	31.0 ± 3.5	.914
Weight,kg	70.3 ± 9.7	66.0 ± 4.9	.127
Height,cm	161.9 ± 6.3	159.7 ± 5.0	.312
Body mass index, kg/m^2^	26.8 ± 3.4	26.0 ± 2.2	.414
Gestational age,wk	39.1 ± 1.0	39.6 ± 1.0	.269
Cervical dilation before analgesia,cm	2 [2-2]	2 [2-2]	.981
NRS before analgesia	8 [6–8]	7 [5–8]	.174

The data are presented as the means ± standard deviations, medians (interquartile ranges).

NRS = numerical rating scale.

**Figure 1.**
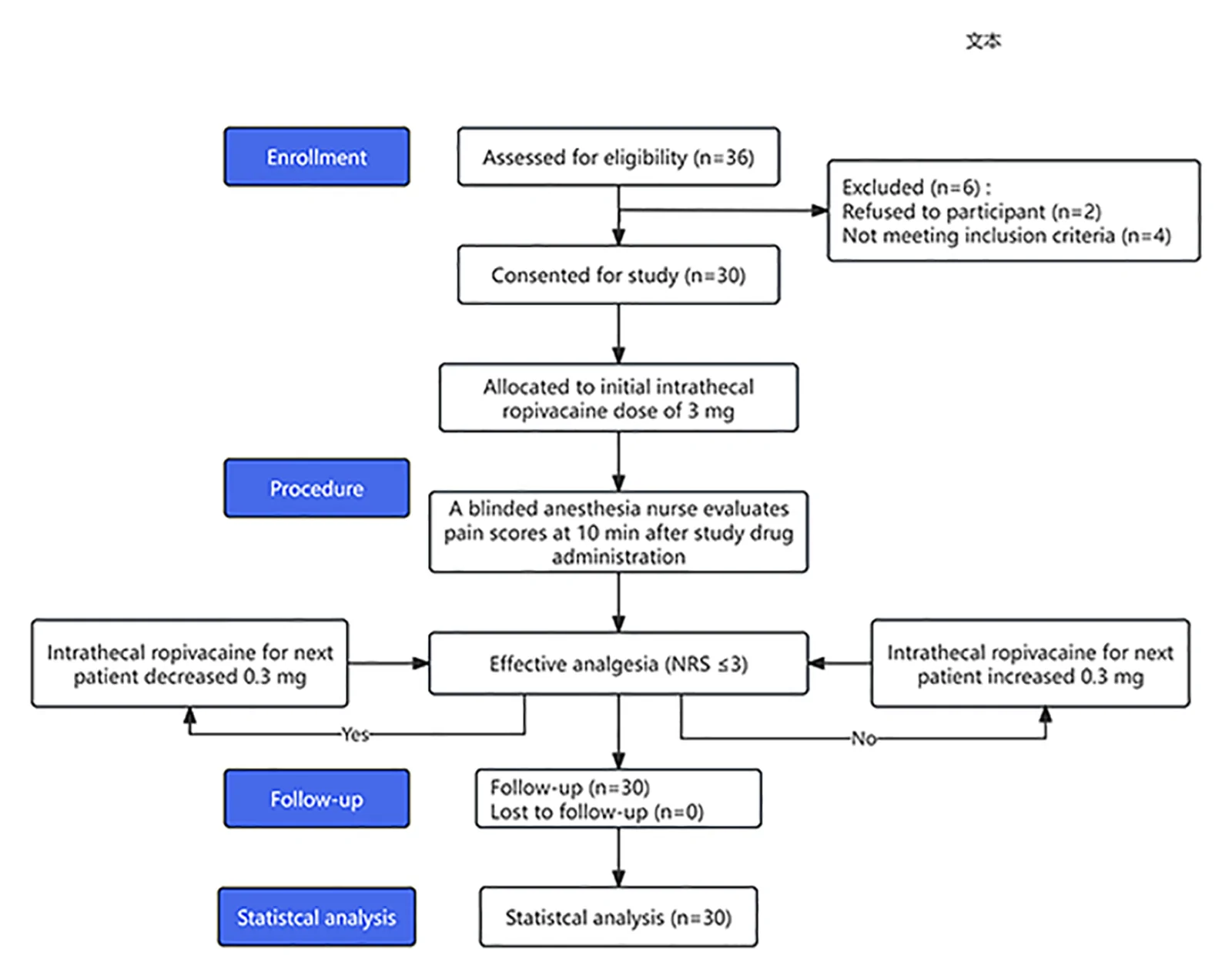
Flow diagram for the up-down method.

### 3.2. ED_50_ and ED_95_ of intrathecal ropivacaine

The ED_50_ and ED_95_ of intrathecal ropivacaine for labor analgesia in multiparous women, estimated via probit regression analysis, were 2.16 mg (95% CI: 1.83–2.39 mg) and 2.76 mg (95% CI: 2.49–3.02 mg), respectively (Figs. 2 and 3). Due to the limited sample size, the precision of the ED_95_ estimate is constrained; therefore, it should be interpreted as a preliminary result. A statistically significant difference in ropivacaine dosage was observed between the effective group and the ineffective group (*P* < .05) (Table 2).

**Table 2:** Maternal and neonatal outcomes.

Variables	Effective group (n = 17)	Ineffective group (n = 13)	*P* value
Ropivacaine dose,mg	2.4 ± 0.3	2.0 ± 0.3	.003
Cesarean delivery, n	1 (3.33%)	0	> .99
Nausea and vomiting,n	0	1 (3.33%)	> .99
Urinary retention,n	1 (3.33%)	1 (3.33%)	> .99
Motor block,n	0	0	/
Hypotension,n	0	0	/
Maternal bradycardia,n	0	0	/
Respiratory depression,n	0	0	/
Abnormal fetal heart rate, n	1 (3.33%)	0	> .99
Apgar 1 min	10 [10,10]	10 [10,10]	1
Apgar 5 min	10 [10,10]	10 [10,10]	1

Data are presented as mean ± standard deviation or number (%) or median with interquartile range [IQR].

**Figure 2.**
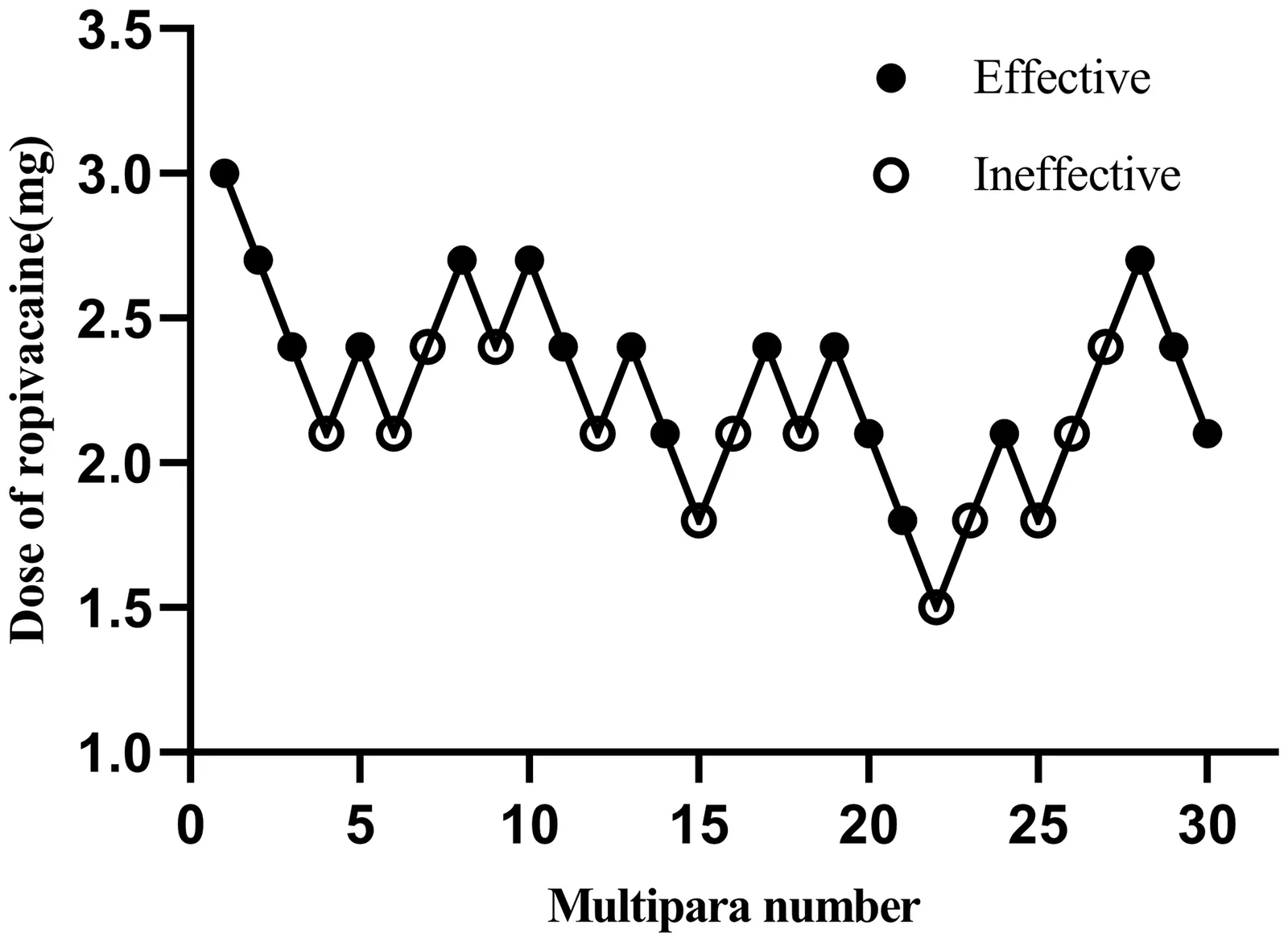
Sequence diagram of intrathecal ropivacaine administration in multiparous women undergoing labor analgesia.

**Figure 3.**
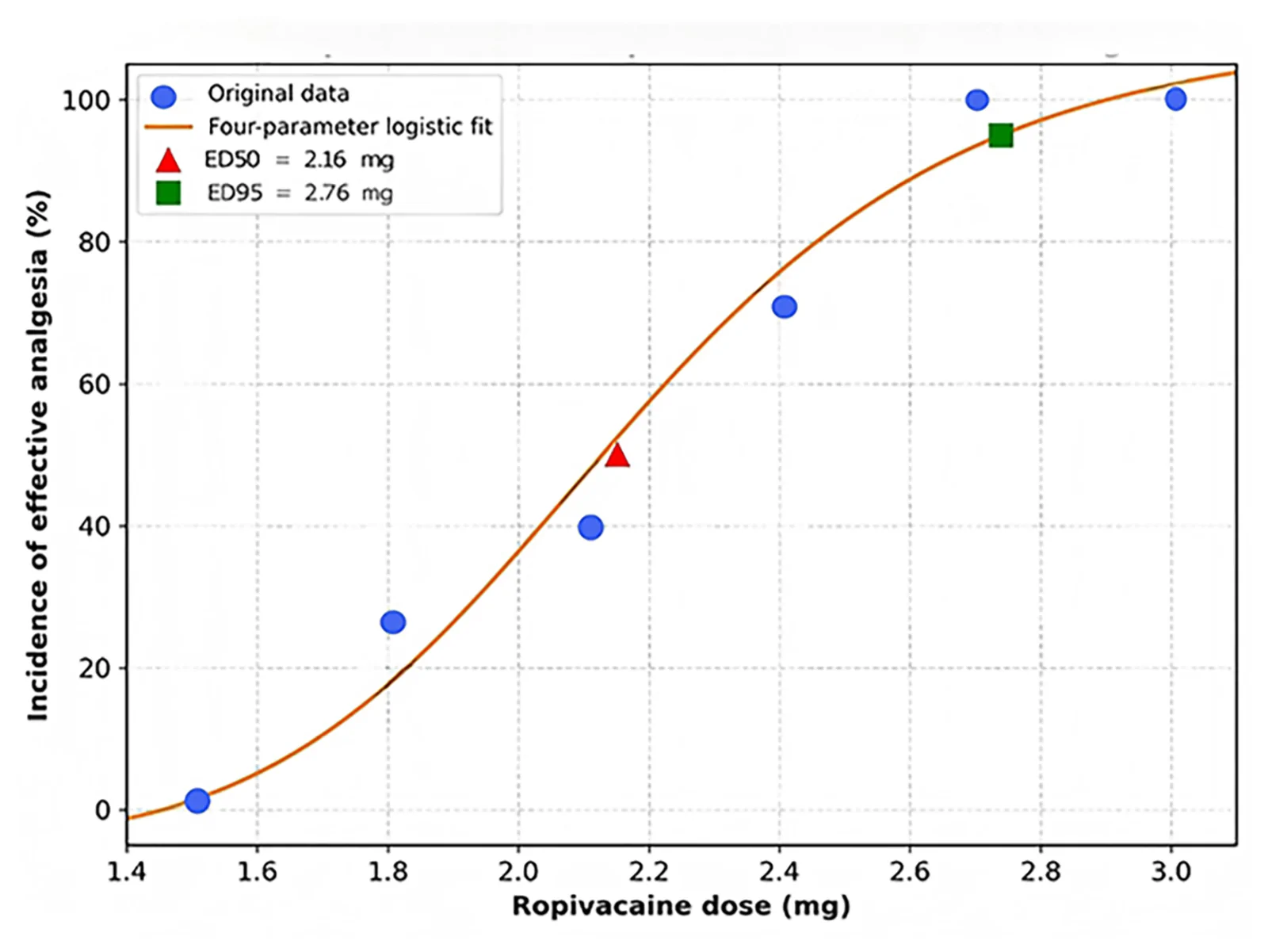
Dose-effect curve of intrathecal ropivacaine administration in multiparous women undergoing labor analgesia.

### 3.3. Maternal and neonatal outcomes

No significant differences in maternal or neonatal outcomes were observed between the 2 groups. In the effective analgesia group, 1 parturient developed fetal bradycardia after analgesia, which resolved with oxygen supplementation and positional adjustment. Nevertheless, recurrent fetal bradycardia during subsequent contractions eventually necessitated cesarean delivery. Another patient in the same group experienced urinary retention. No cases of bradycardia, respiratory depression, hypotension, or motor block were recorded in either group (Table 2).

## 4. Discussion

This study determined the ED_50_ and ED_95_ of intrathecal ropivacaine for labor analgesia in multiparous women to be 2.16 mg (95% CI: 1.83–2.39 mg) and 2.76 mg (95% CI: 2.49–3.02 mg), respectively. Maternal adverse effects were infrequent, and all neonates remained safe. Within a sequential allocation design, dose-response relationships of relevant clinical parameters were systematically evaluated. These findings contribute to bridging the current evidence gap concerning intrathecal analgesia in multiparous women during the spontaneous latent phase of labor and support its safe and rational application in clinical anesthetic practice.

Among the 30 patients, effective analgesia was achieved in 17 (56.7%), while 13 (43.3%) experienced ineffective analgesia. The 2 groups were comparable in demographic and obstetric characteristics, helping to rule out patient-related factors as a cause of the inadequate spinal analgesia – a recognized contributor to inadequate neuraxial anesthesia.^[15]^

Ropivacaine is widely employed for neuraxial labor analgesia owing to its favorable motor-sparing properties and hemodynamic stability. However, low doses may result in inadequate sensory blockade, while high doses can induce motor impairment despite effective pain relief. The typical intrathecal dose of ropivacaine for labor analgesia ranges from 2.5 to 3 mg.^[4,9]^ For instance, Li et al administered 5 mg ropivacaine with 2.5 μg sufentanil intrathecally, achieving 100% onset of warmth and numbness within 3 minutes; however, 77.55% of parturients developed motor blockade, indicating probable drug overdose.^[16]^ In another study, Yin et al reported that 2.5 mg ropivacaine combined with 2.61 μg (95% CI, 2.44–2.70 μg) sufentanil provided faster analgesia onset compared with conventional epidural techniques, yet 77.5% of participants experienced pruritus.^[5]^ To avoid opioid-related side effects, the present study used ropivacaine intrathecally alone without adjuvant sufentanil.

Ngan et al reported an ED_50_ of 1.95 mg for intrathecal ropivacaine in nulliparous women.^[11]^ In contrast, our study determined the ED_50_ to be 2.16 mg in multiparous women, which is higher than that in nulliparous women. This result appears to challenge the conventional clinical assumption that nulliparous women experience more intense labor pain than multiparous women, a view often influenced by clinical experience and potential bias.^[17,18]^ However, a growing body of research aligns with and contextualizes our findings. Previous studies have noted that negative birth events such as cesarean sections, episiotomies, or instrumental deliveries may similarly elevate the risk of birth-related fear in multiparous women.^[19]^ Agaram et al reported that cervical dilation > 7 cm, a history of opioid tolerance, and a previous failed epidural are associated with higher odds of inadequate pain relief.^[20]^ In a similar vein, Peng Q et al observed that multiparous women exhibited an increased need for analgesia, which may be linked to psychogenic pain components reinforced by memories of prior labor.^[12]^ Additionally, multiparous women generally experience stronger uterine contractions and may develop heightened sensitivity to nociceptive stimuli, possibly modulated by the recollection of previous labor pain.^[1]^

Compared with epidural labor analgesia, combined spinal‑epidural analgesia has been associated with a higher incidence of non‑reassuring fetal heart rate patterns.^[21]^ Previous literature reports an incidence of approximately 12.5%, though most cases were transient and resolved spontaneously.^[5]^ Notably, those studies included intrathecal sufentanil as an adjuvant. In the present study, one case of non‑reassuring fetal heart rate was observed; however, it resolved promptly with oxygen supplementation and positional adjustment. The subsequent cesarean delivery in this case was due to cord‑related complications later in labor, rather than the intrathecal medication. The absence of hypotension observed in our study is likely attributable to the ultra-low dose of intrathecal ropivacaine and the omission of adjunctive sufentanil, compounded by the physiological distinction that laboring parturients maintained normal oral intake, unlike the fasted state typical of cesarean section patients. Although the limited sample size precludes definitive causal conclusions, this favorable safety profile supports the feasibility of this regimen and warrants further investigation in multiparous women.

Several limitations of this study should be acknowledged. First, the up‑and‑down method is primarily intended for estimating the ED_50_, and the derived ED_95_ should therefore be interpreted with caution. Future studies employing a biased‑coin design or a larger sample analyzed with probit regression over a broader dose range are needed to determine the ED_95_ with greater precision. Second, the sequential allocation design may have been influenced by obstetric factors such as initial cervical dilation, inter‑individual variability in pain perception, and pre‑analgesia NRS scores, all of which can affect analgesic efficacy. Third, the findings mainly reflect the characteristics of multiparous women in spontaneous latent‑phase labor and may not generalize to those in active‑phase labor, which is typically associated with greater pain intensity and potentially higher ropivacaine requirements. Fourth, the volume and dose of local anesthetics are critical determinants of spinal analgesia efficacy. We used a 3 mL spinal dose, aligning with standard cesarean delivery practice to ensure adequate block depth. Nevertheless, lower volumes (<3 mL) might suffice for labor analgesia, warranting further investigation to establish the optimal dosing. Finally, the exclusion of parturients with minimal pain or those who delivered before analgesia initiation could introduce selection bias, which may limit the broader applicability of the results.

## 5. Conclusion

In conclusion, this study preliminarily determined that the ED_50_ of intrathecal ropivacaine for labor analgesia in multiparous women during spontaneous labor in the latent phase was 2.16 mg. The ED_95_ estimated by model extrapolation was 2.76 mg, which may serve as a reference for future studies; however, its accuracy requires further confirmation in larger-scale investigations.

## Acknowledgments

We sincerely thank all the staff of the Department of Anesthesiology, Fujian Maternity and Child Health Hospital, for their support in conducting this study, as well as all the multiparous women who participated.

## Author contributions

**Project administration:** SuJing Zhang, PeiHan Xiao.

**Validation:** SuJing Zhang, ZhiRong Yan.

**Writing – review & editing:** SuJing Zhang, LongXin Zhang.

**Data curation:** ZhiRong Yan.

**Writing – original draft:** ZhiRong Yan, PeiHan Xiao.

**Conceptualization:** PeiHan Xiao, LongXin Zhang.

**Funding acquisition:** PeiHan Xiao.

**Investigation:** LiYan Miao, XiaoFen Chen.

**Methodology:** LiYan Miao, XiaoFen Chen.

**Supervision:** LongXin Zhang.
